# Supplementary material for: Effect of the COVID-19 pandemic on the care for acute cholecystitis: a Swedish multicentre retrospective cohort study
Source: BMJ Open. 2023 Nov 30;13(11):e078407. doi: 10.1136/bmjopen-2023-078407 (PMC10689379; doi:10.1136/bmjopen-2023-078407)
Supplement: Author's manuscript [file bmjopen-2023-078407.draft_revisions.pdf]

BMJ Open

BMJ Open is committed to open peer review. As part of this commitment we make the peer review history of every article we publish publicly available.

When an article is published we post the peer reviewers' comments and the authors' responses online. We also post the versions of the paper that were used during peer review. These are the versions that the peer review comments apply to.

The versions of the paper that follow are the versions that were submitted during the peer review process. They are not the versions of record or the final published versions. They should not be cited or distributed as the published version of this manuscript.

BMJ Open is an open access journal and the full, final, typeset and author-corrected version of record of the manuscript is available on our site with no access controls, subscription charges or pay-per-view fees (http://bmjopen.bmj.com).

If you have any questions on BMJ Open's open peer review process please email info.bmjopen@bmj.com

Effect of the COVID-19 Pandemic on the Care for Acute Cholecystitis – a Multicenter Retrospective Cohort Study

Journal:	*BMJ Open*
Manuscript ID	bmjopen-2023-078407
Article Type:	Original research
Date Submitted by the Author:	01-Aug-2023
Complete List of Authors:	Osterman, Erik; Uppsala University, CKF Gävleborg; Gävle sjukhus, Department of Surgery<br>Jakobsson, Sofia; Uppsala University, Department of Surgical Sciences<br>Larsson, Christina; Gävle sjukhus, Department of Surgery<br>Linder, Fredrik; Uppsala University Hospital, Department of Surgery; Uppsala University, Department of Surgical Sciences
Keywords:	COVID-19, Observational Study, Retrospective Studies, SURGERY

SCHOLARONE™
Manuscripts

I, the Submitting Author has the right to grant and does grant on behalf of all authors of the Work (as defined in the below author licence), an exclusive licence and/or a non-exclusive licence for contributions from authors who are: i) UK Crown employees; ii) where BMJ has agreed a CC-BY licence shall apply, and/or iii) in accordance with the terms applicable for US Federal Government officers or employees acting as part of their official duties; on a worldwide, perpetual, irrevocable, royalty-free basis to BMJ Publishing Group Ltd ("BMJ") its licensees and where the relevant Journal is co-owned by BMJ to the co-owners of the Journal, to publish the Work in this journal and any other BMJ products and to exploit all rights, as set out in our licence.

The Submitting Author accepts and understands that any supply made under these terms is made by BMJ to the Submitting Author unless you are acting as an employee on behalf of your employer or a postgraduate student of an affiliated institution which is paying any applicable article publishing charge ("APC") for Open Access articles. Where the Submitting Author wishes to make the Work available on an Open Access basis (and intends to pay the relevant APC), the terms of reuse of such Open Access shall be governed by a Creative Commons licence – details of these licences and which Creative Commons licence will apply to this Work are set out in our licence referred to above.

Other than as permitted in any relevant BMJ Author's Self Archiving Policies, I confirm this Work has not been accepted for publication elsewhere, is not being considered for publication elsewhere and does not duplicate material already published. I confirm all authors consent to publication of this Work and authorise the granting of this licence.

For peer review only

Effect of the COVID-19 Pandemic on the Care for Acute Cholecystitis – a Multicenter Retrospective Cohort Study

Authors

Erik Osterman MD PhD [1,4] *† (ORCID 0000-0003-1621-7872), Sofia Jakobsson[3] (ORCID 0009-0003-2648-6213), Christina Larsson MD[1], Fredrik Linder MD PhD [2,3] (ORCID 0000-0001-8125-4536)

Affiliations

1 Department of Surgery, Gävle Hospital, Gävleborg Region, Sweden

2 Department of Surgery, Uppsala University Hospital, Uppsala Region, Sweden

3 Department of Surgical Sciences, Uppsala University, Sweden

4 Centre for Clinical Research Gävleborg, Uppsala University, Sweden

*Corresponding author

† Contributed equally to this paper

Corresponding author: Erik Osterman, Department of Surgery, Gävle Hospital, Region Gävleborg 80187 Sweden

erik.osterman@igp.uu.se erik.osterman@regiongavleborg.se

Cell: +46735838735. Work: +4626157685

Manuscript type: Original Article

Word count: 2 992 + Tables 505 words + Statements 309 words

Abstract: 241

For peer review only

ABSTRACT

Objectives: The present study aimed to investigate if and how the panorama of acute cholecystitis changed during 2020 at three Swedish centres in two counties. Seven aspects were identified, the incidence of cholecystitis, the Tokyo grade, the timing of diagnosis and treatment, the proportion treated with early surgery, the proportion of patients treated with delayed surgery, and new complications from gallstones.

Design: Retrospective multicenter cohort study

Setting: 3 hospitals in Sweden, covering 675,000 inhabitants

Participants: 1,634 patients with cholecystitis

Outcomes: The incidence, treatment choice and diagnostic and treatment delay were investigated by comparing pre-pandemic and pandemic patients.

Results: Patients diagnosed with cholecystitis during the pandemic were more comorbid (ASA2-5 86% vs 81%, p 0.01) and more often had a diagnostic computed tomography (67% vs 59% p 0.01). There were variations in the number of patients corresponding with the pandemic waves, but there was no overall increase in the number of patients with cholecystitis or the proportion of patients treated with surgery during the pandemic. There was no increase in time to admission or surgery from admission (median 2 and 1 days), and the proportion of grade 2-3 cholecystitis was not higher. The median time to elective surgery increased (184 days vs 130 days, p 0.04), but there was no increase in new gallstone complications.

Conclusion: Emergency surgery for cholecystitis was not impacted by the pandemic. Patients were more comorbid but did not have a more severe grade of cholecystitis nor was there a delay in seeking care. Fewer patients non-operatively managed had elective surgery within 6 months of their initial diagnosis but there was no corresponding increase in gallstone complications.

For peer review only - http://bmjopen.bmj.com/site/about/guidelines.xhtml

Strengths and limitations of this study

- Covers all patients seeking hospital care for cholecystitis
- Utilizes both electronic patient records and prospectively collected registry data
- Any patient solely cared for in primary care not included
- Only includes 2020 as a pandemic year
- Mainly Retrospective study

For peer review only

For peer review only - http://bmjopen.bmj.com/site/about/guidelines.xhtml

BACKGROUND

Early cholecystectomy is the recommended treatment for acute cholecystitis. Non-operatively managed (NOM) patients may be considered for surgery later to avoid new complications from cholecystolithiasis.[1] During the coronavirus disease 2019 (COVID-19) pandemic, some elective surgical procedures were deferred to handle the effects of the pandemic.[2] The pandemic affected uncomplicated cholecystectomies more than other procedures[3,4], and the number of elective cholecystectomies in Sweden decreased by 30% while emergency procedures only decreased by 1%.[5] In 2020 the pandemic hit Sweden in two waves, the first starting in March and the second in October.[5] Sweden had less strict policies to stop the spread of the virus than many other countries.[6]

Due to concerns over of aerosolized virus in asymptomatic patients open surgery was preferred initially.[7] This might have resulted in fewer emergency cholecystectomies, as postoperative complications are more common, especially in obese patients. In combination with reduced capacity for elective cholecystectomies, the concern was a possible increase in the number of recurrent gallstone complications.

Previous studies

Increased severity of cholecystitis and fewer patients being treated with early cholecystectomy for cholecystitis have been seen during the COVID-19 pandemic.[8,9] The Swedish Registry of Gallstone Surgery and Endoscopic Retrograde Cholangiopancreatography (Gallriks)[10] reported fewer elective procedures while emergency procedures increased, but no data were available on non-operative management.[11] The incidence of cholecystitis during the pandemic has not been completely investigated, cholecystitis decreased in the US[12] while it remained the same in

For peer review only - http://bmjopen.bmj.com/site/about/guidelines.xhtml

Canada,[13] increased in Germany[9] and CHOLECOVID[8] only showed a decrease in mild cholecystitis. Emergency department visits fell in many places [5,12,13] which could indicate that patients waited at home and then presented after 5-7 days when conservative treatment is recommended explaining falling emergency surgery numbers. However, a German study investigated the proportion of non-surgically treated patients and found that non-operative management did not increase during the pandemic.[14]

Aims and hypothesis

The present study aimed to investigate if and how the panorama of acute cholecystitis changed during 2020 at three Swedish centres in two counties. Seven aspects were identified, the incidence of cholecystitis, the Tokyo grade,[15] the timing of diagnosis and treatment, the proportion treated with early surgery, the proportion of patients treated with delayed surgery, and new complications from gallstones. The hypothesis was that the incidence of cholecystitis and the proportion treated with early surgery did not change while the grade increased, and patients were diagnosed later. We also hypothesized that fewer non-operatively treated patients had surgery within 6 months of discharge and more patients had new gallstone complications if diagnosed in 2020.

For peer review only

METHOD

The Swedish Ethical Review Authority approved the study, number 2021-00862. The manuscript was prepared following the Reporting of studies Conducted using Observational Routinely-collected Data (RECORD) checklist.[16] Patients or the public were not involved in the design, or conduct, or reporting, or dissemination plans of our research.

Patients with a diagnosis of acute cholecystitis between 2017-2020 were identified by the administrative data offices of Region Gävleborg and Region Uppsala. The data was then gathered from electronic patient records (EPR) and Gallriks as described previously.[17] The chart review started in mid-2021 and finished in early 2022. The possibility of chart review for the study staff determined the choice of regions.

The outcomes were: Incidence of cholecystitis (number of cases/100,000 inhabitants), grade (G1: mild or G2-3: moderate/severe),[15] the time to first contact with the emergency department and diagnosis from symptoms in days, time to surgery, treatment choice (surgery or NOM), time to delayed surgery, the proportion NOM patients who underwent surgery within 6 months and new gallstone complication after discharge. Exposure was a diagnosis of acute cholecystitis during the pandemic (after March 13 2020).[18] Patients were stratified by diagnosis time, pre-pandemic or pandemic to analyze outcomes and demographics. To assess delays in delayed surgery all patients diagnosed during 2020 were analyzed as the exposed group. The number of inhabitants over 18 years of age in the two regions was obtained from Statistics Sweden and was transformed to person-years of exposure. The results were then checked against the national patient registry to further investigate the incidence of cholecystitis.

For peer review only - http://bmjopen.bmj.com/site/about/guidelines.xhtml

Statistical analysis

Differences between groups were tested with the χ^2 test for categorical variables and the Wilcoxon rank sum test for discrete variables. Post-hoc testing with Holm correction for multiple testing was used for variables significant with more than two levels. The incidence was analyzed in two ways, first calculated from the monthly rate and adjusted for the population size of the counties, and second by also adjusting for seasonality with the X11 procedure.[19] Logistic regression was used to analyze the proportion of NOM patients undergoing elective surgery within 6 months of their cholecystitis adjusting for comorbidities. A Cox proportional hazards model was used to investigate the time new gallstone complications censoring for death and surgery. The number of patients each month and the proportion having surgery compared to the previous period was plotted. The monthly number of ICU admissions for COVID-19 was overlayed to convey the waves of the pandemic, the data came from the Swedish Intensive Care Registry.[20] Data was not imputed, and a complete case analysis was performed where applicable. Power analysis for an effect size of 0.3 with 95% power and α 0.05, for the Wilcoxon rank sum test and χ^2 test with 4 degrees of freedom gave a required sample of at least 854 and 207 patients which determined the number of control years to include in the study. Logistic regression required a sample size of 636 to detect a 25% decrease in elective surgery within 6 months at 95% power and α 0.05. The Cox proportional hazards regression required a sample size of 954 to detect an increase of 50% (HR 1.5) in new complications at 95% power and α 0.05. Statistics were calculated with R version 4.2.2 (Vienna, Austria).

For peer review only - http://bmjopen.bmj.com/site/about/guidelines.xhtml

RESULTS

In total, 1,634 patients were included in the study, 1,286 patients were diagnosed before the pandemic and 348 after the start of the pandemic. The incidence of first-time cholecystitis did not change during the pandemic (76/100,000 cases/person-years pre-pandemic vs 81/100,000 cases/person-years, p 0.3, χ^2 test, data in table 1). Adjusting for seasonality did not change the result. The national patient registry did not show a significant change in the incidence of cholecystitis between the years 2017-2019 and 2020-2021 either.

There was no difference in sex, age, or BMI between the two periods, table 2. During the pandemic, there was a 13% increase in patients classified as 2 in the American Society of Anesthesiologists physical classification system (ASA).[21] There was a corresponding 22% decrease in otherwise healthy patients (posthoc test ASA1 p 0.23 and ASA2 p 0.08). More patients had computed tomography (CT) as a diagnostic modality during the pandemic (59% pre-pandemic vs 67%, post-hoc test p 0.007). Five patients had concurrent COVID-19.

Table 1: Incidence of new cholecystitis 2017-2020, excluding recurrences during that period.

	Period	Person-years	Cases	Cases/100,000	p
Unadjusted	***Pre-pandemic***	1,685,729	1286	76	0.3
	Pandemic *(From 2020-03-13)*	429,600	348	81	
Seasonally adjusted	***Pre-pandemic***	1,713,119	1297	76	0.7
	Pandemic *(From 2020-04-01)*	429,600	313	78	

P value calculated with χ^2 test, only persons ≥18 years old used for person-years.

For peer review only

Unchanged grade and admission times

There was no difference in the proportion of grade 1 versus grade 2-3 cholecystitis between the two periods, table 2. No difference in leukocyte counts and C-reactive protein at admission was noted suggesting that patients were not admitted later in the disease progression (median leukocyte count 12.3x10^9/l pre-pandemic vs 12.3x10^9/l, p 0.53, median CRP 74 mg/l pre-pandemic vs 83 mg/l, p 0.62, Wilcoxon rank sum test). This was confirmed by a lack of difference in time to first contact with the emergency department and admission after symptoms. No difference in time to surgery was noted either suggesting that patients treated with surgery had the same priority as before the pandemic. There was no difference in the length of stay for patients treated with surgery (median 3 days, p 0.75) or without surgery (median 4 days, p 0.95).

Treatment choice

There was no difference in the proportion of patients treated with early surgery during the pandemic (44% pre-pandemic vs 46%, p 0.41), table 2. There was no difference in the proportion of laparoscopic (38% pre-pandemic vs 38%), open (4% pre-pandemic vs 5%) and surgeries converted to open (2% pre-pandemic vs 3%) between the two periods (p 0.14). For patients NOM there was no difference in the proportion receiving drains or the plan for follow-up, 24% of patients were planned for delayed surgery either at discharge or after a visit to the out-patient clinic (p 0.24). Surprisingly there was no difference in the proportion of patients referred to the outpatient clinic during the pandemic with 38% of NOM patients being referred and about 40% of those being planned for surgery during both periods (p 0.45).

For peer review only - http://bmjopen.bmj.com/site/about/guidelines.xhtml

185

For peer review only

For peer review only - http://bmjopen.bmj.com/site/about/guidelines.xhtml

Table 2: Demographics, treatment and outcomes for patients before and during the COVID-19 pandemic.

	Total	Pre-pandemic	Pandemic	p
**Sex**				
Male	857 (52%)	666 (52%)	191 (55%)	0.33
Female	777 (48%)	620 (48%)	157 (45%)	
**Age**				
Median (IQR)	67.2 (51.1 - 77.0)	67.2 (50.7 - 76.8)	67.2 (53.9 - 77.5)	0.27
**BMI**				
Median (IQR)	27.8 (24.7 - 31.4)	27.8 (24.7 - 31.3)	27.9 (24.7 - 31.5)	0.98
Missing	117 (7.2%)	93 (7.2%)	24 (6.9%)	
**ASA**				
1	300 (18%)	250 (19%)	50 (14%)	0.013
2	731 (45%)	554 (43%)	177 (51%)	
3	515 (32%)	405 (31%)	110 (32%)	
4	87 (5%)	76 (6%)	11 (3%)	
5	1 (0%)	1 (0%)	0 (0%)	
**CCI**				
Median (IQR)	3.0 (1.0 - 5.0)	3.0 (1.0 - 5.0)	3.0 (1.0 - 5.0)	0.43
**Previous stone**				
No	1,176 (72%)	919 (71%)	257 (74%)	0.42
Yes	458 (28%)	367 (29%)	91 (26%)	
**Imaging**				
No radiology	59 (4%)	50 (4%)	9 (3%)	0.01
US	579 (35%)	475 (37%)	104 (30%)	
CT	570 (35%)	424 (33%)	146 (42%)	
CT+US	424 (26%)	336 (26%)	88 (25%)	
Other+MRCP	2 (0%)	1 (0%)	1 (0%)	
**Cholecystitis Grade**				
G1	798 (49%)	627 (49%)	171 (49%)	0.85
G2-3	729 (45%)	569 (44%)	160 (46%)	
Missing	107 (7%)	90 (7%)	17 (5%)	
**Treatment**				
Conservative	815 (50%)	642 (50%)	173 (50%)	0.41
Surgery	725 (44%)	565 (44%)	160 (46%)	
Drain	94 (6%)	79 (6%)	15 (4%)	
**Time to first contact from symptoms**				
Median (IQR)	1.0 (0.0 - 3.0)	1.0 (0.0 - 3.0)	1.0 (0.0 - 3.0)	0.78
Missing	57 (3.5%)	45 (3.5%)	12 (3.4%)	
**Time to admission from symptoms**				
Median (IQR)	2.0 (1.0 - 3.0)	2.0 (1.0 - 3.0)	2.0 (1.0 - 3.0)	0.7
Missing	107 (6.5%)	89 (6.9%)	18 (5.2%)	
**Time to surgery from admission**				
Median (IQR)	1.0 (1.0 - 2.0)	1.0 (1.0 - 2.0)	1.0 (1.0 - 2.0)	0.86
**Length of stay (days)**				
Median (IQR)	3.0 (2.0 - 6.0)	3.0 (2.0 - 6.0)	3.0 (2.0 - 5.0)	0.79
Missing	52 (3.2%)	45 (3.5%)	7 (2.0%)	
**Plan for follow-up**				
No	829 (73%)	657 (72%)	172 (76%)	0.24
Surgery	304 (27%)	251 (28%)	53 (24%)	
**Readmitted within 30 days**				
No	1,445 (88%)	1,138 (88%)	307 (88%)	0.92
Yes	189 (12%)	148 (12%)	41 (12%)	

Fisher exact test used for categorical variables, Wilcoxon rank sum test for discrete variables.
ASA: American Society of Anesthesiologists Performance Status Classification, BMI: Body Mass Index, CCI: Charlson Comorbidity Index. IQR: Interquartile range. US: Ultrasound

For peer review only

For peer review only - http://bmjopen.bmj.com/site/about/guidelines.xhtml

Temporal trends

The number of cases and proportion treated with early surgery compared with the three previous years was calculated to investigate if the pandemic changed the number of patients and the treatment choice month by month, figure 1. During the first three months of the year, there were approximately 20% more patients than the previous years and the proportion treated with early surgery was higher, in April as the pandemic hit the total cases went down and a higher proportion of patients had surgery. During the second wave starting in October, the proportion treated with early surgery fell while cases remained comparable to 2017-2019 numbers. Changes were not statistically significant, and the figure is meant to illustrate the changes that were seen during the pandemic.

Delayed surgery and new gallstone complications

The Tokyo guidelines consider delayed surgery to be surgery performed at least 42 days after acute cholecystitis.[1] The median time to surgery in the two regions in 2017-2019 was 130 days (interquartile range, IQR 76, 214) while it was 184 days (IQR 78,341) in 2020 (p 0.04 Wilcoxon rank sum test). In 2017-2019 20% of NOM patients had surgery within 6 months while in 2020 only 11% had surgery within this time (p 0.003, χ^2 test). The only patient variable differing between the periods was ASA classification, and since this might affect the proportion treated with early surgery it was included in the logistic regression, table 3. Adjusted odds ratio (OR) for surgery within 6 months in NOM patients was 0.47 (95%CI 0.29-0.74, p 0.002). This is in line with the longer median time to surgery seen in the unadjusted analysis.

The risk of new gallstone complications was not impacted by this as 39% of patients NOM in 2017-2019 and 35% in 2020 had recurrent gallstone disease (p 0.3, χ^2test). There was no change in the pattern of new gallstone complications with new cholecystitis in 140 of 721

For peer review only

For peer review only - http://bmjopen.bmj.com/site/about/guidelines.xhtml

patients pre-pandemic (19%) and 33 of 188 (18%) patients during the pandemic. Cholangitis was the first complication in 14 patients (2%) pre-pandemic and 6 patients (3%) during the pandemic. Pancreatitis was the first complication in 11 patients (2%) pre-pandemic and 5 patients (3%) during the pandemic. The median time to new gallstone complication was 87 days (IQR 29-249) pre-pandemic and 76 days (IQR 23-194) during the pandemic (n.s.). Accounting for the follow-up with Cox proportional hazards model confirmed that there was no difference, HR 0.91 (95%CI 0.67-1.22, p 0.5).

Table 3: Logistic regression for delayed surgery within 6 months

**Characteristic**	**OR**[1]	**95% CI**[1]	**p-value**
**Year**			
**2017-2019**	—	—	
**2020**	0.47	0.29, 0.74	0.002
**ASA**			
**1**	—	—	
**2**	0.72	0.45, 1.17	0.2
**3**	0.22	0.13, 0.38	<0.001
**4**	0.05	0.01, 0.16	<0.001

[1] OR = Odds Ratio, CI = Confidence Interval

For peer review only

For peer review only - http://bmjopen.bmj.com/site/about/guidelines.xhtml

DISCUSSION

During the first month of the pandemic, there were fewer patients seeking hospital care for cholecystitis, which was compensated by more patients later in the year. Overall, there was no difference in the incidence of first-time cholecystitis during the pandemic. The proportion of patients who had early surgery remained the same over time, but some months had a higher proportion of early surgery than previous years, despite patients diagnosed during the pandemic being more comorbid. This might have been a result of knowing that resources for delayed surgery were scarce, as shown in this paper and previous research from Gallriks. [11]

The differences in ASA classification between the periods could be from otherwise healthy patients staying at home, thinking they had COVID-19. Testing capacity was limited at the beginning of 2020 thus abdominal pain and fever might have been considered COVID-19 instead of cholecystitis. Chest CT was sometimes performed instead of a nasopharyngeal swab which could explain the higher proportion of CT use.[22] Limited testing may also explain the low number of patients with concurrent COVID-19 infection. Another reason might be that ultrasound (US) was avoided to prevent close contact and transmission of infection. A third reason for the increase in CT use could be the limited availability of hospital beds, which could have led to CT being used as a tool to decide which patients needed to be admitted instead of waiting for further tests and US.

Comorbid patients might be more likely to seek out healthcare, especially if fearing that they had contracted a potentially deadly infection. This is similar to what the CHOLECOVID collaboration found, patients were sicker during the pandemic.[8] Another possibility is that the documentation of comorbidities got better during the pandemic. However, the CCI was unchanged, suggesting that it were diseases not included in the CCI that led to ASA1 patients

For peer review only - http://bmjopen.bmj.com/site/about/guidelines.xhtml

being classified as ASA2, e.g. hypertension or smoking. Two studies have compared the documentation of code status finding that it improved in a UK hospital (from a low level) and found no change in a Dutch hospital (already at a high level).[23,24] However, the documentation of comorbidities has not been investigated in any study.

There was no increase in the grade of cholecystitis and the patients that did seek care did not do so later and were admitted and operated upon in a similar time frame as the previous three years, which was not investigated by CHOLECOVID or the national Swedish study.[8,11] There were no other indications that patients presented later, e.g. the leukocyte count or CRP. Emergency surgical care for patients with cholecystitis does not seem to have been affected by the pandemic in Sweden. If this is the result of universal healthcare in contrast with the US or from more relaxed policies in contrast with Germany and Canada is not entirely clear.[12,13] The incidence and proportion of operatively managed patients did not decrease in Canada, which has universal healthcare but had tougher policies initially. In the US there was a demographic shift towards younger and privately insured patients compared to the time before the pandemic.[12] In Germany, with the strictest policies of all, the incidence of cholecystitis rose.[9] However, it is not entirely comparable to Sweden since it runs an insurance-based healthcare system with an abundance of hospital beds.[25]

For peer review only

The follow-up and subsequent delayed surgery for NOM patients were further delayed. This is in line with guidelines at the time prioritizing emergency surgery and malignancy and previous research.[2,11] The median wait time for delayed cholecystectomy was too long before the pandemic and the limited resources of the pandemic increased it by almost two months. Despite this, there was no increase in recurrent gallstone complications when adjusting for follow-up of patients diagnosed during the pandemic. Previous research in the

For peer review only - http://bmjopen.bmj.com/site/about/guidelines.xhtml

same population highlighted that recurrent gallstone disease is most common in the first months after discharge and common in NOM patients.[17] Thus, delayed surgery did not stop them previously, and delaying it further did not change this.

Strengths and limitations

One of the main strengths of the study is the inclusion of all patients, not only those undergoing surgery. Patients cared for by general practitioners would be missing from the analysis. Based on our clinical experience, most of these patients were previously referred to the hospital if they had a fever and abdominal pain. However, they may not have been referred during the pandemic if they were otherwise healthy or thought to have abdominal symptoms from COVID-19.

This study included patients throughout the entirety of 2020 compared with CHOLECOVID which only included patients from the first two months of the pandemic. The risk of selection bias is lower than in a prospective study though with the drawbacks of a retrospective study where inclusion was dependent on correct classification in the EPR. Standardization of data collection was attempted and by utilizing EPR data recall bias is limited. However, some notes lacked detail. The data is likely representative of countries with similar healthcare systems and pandemic responses. Sensitivity analysis gave a required effect size of 0.22 for the Wilcoxon Ranked sum test to detect a difference at 95% power and α 0.05 and 0.1 for the X^2 test to detect a difference at 4 degrees of freedom. The logistic regression was sufficiently powered, while the Cox proportional hazards regression was powered to detect an increase of 50% in complications, thus smaller differences are not ruled out.

For peer review only

The study only investigated the treatment for the first cholecystitis in the period 2017-2020 and subsequent events were classified as new gallstone complications. Patients with their first cholecystitis at the end of 2019 would also be impacted by the pandemic and a reduced capacity for elective surgery in 2020. The subsequent waves in 2021 were not included in this study thus the study is limited to the initial response and changed policies. The pandemic group does not include the first two months of the year, where fewer patients have cholecystitis due to seasonal variation. The comparisons with previous years were performed on a month-to-month basis and the incidence was adjusted for season to account for this.[26]

Future perspectives

This study investigated the first cholecystitis in patients during the period 2017-2020 and did not find a change in the incidence. While there was no increased incidence of cholecystitis in the present study, other emergency general surgery diseases were affected by the pandemic, the incidence of appendicitis increased according to Swedish studies, potentially as a direct effect of COVID-19 infection.[27] The national patient registry which contains data on diagnoses and surgical treatment could allow for the evaluation of a national material and cover other EGS diseases besides cholecystitis. It would be especially interesting to investigate emergency general surgery diseases that are usually treated without surgery or antibiotics, e.g. diverticulitis, to assess if patients stayed away from the hospitals during the pandemic.

Conclusion

At our centres, emergency surgery for cholecystitis was not impacted by the pandemic. Patients were more comorbid but did not have a more severe grade of cholecystitis nor did

For peer review only

For peer review only - http://bmjopen.bmj.com/site/about/guidelines.xhtml

they seek care later. Fewer patients NOM had elective surgery within 6 months of their initial diagnosis but there was no corresponding increase in gallstone complications.

STATEMENTS, DISCLOSURES, ETC.

Acknowledgements

We want to thank the EPR and analysis departments at Region Gävleborg and Region Uppsala for their help with identifying the population from ICD codes. We also want the thank the surgeons and administrators reporting to Gallriks for making the study possible. Thanks to Anders Blomberg and Jennie Wickenberg for discussions regarding the design of the study. Thanks to Louise Helenius and Tamali Majumder for helping with the collection of data.

Disclosure, Competing interests

No conflicts of interest.

Funding

The research was funded by the Centre for Research and Development, Gävleborg Region. EO, LH, CL, AB, JW are funded by Region Gävleborg. FL was financed by grants from the Swedish state under the agreement between the Swedish Government and the Uppsala County Council (ALF). Open access funding from Uppsala University.

Availability of data and materials

The data are not publicly available due to information that could compromise the privacy of research participants. The registry data that support the findings of this study are available from Gallriks (https://www.ucr.uu.se/gallriks/). Restrictions apply to the availability of these

For peer review only - http://bmjopen.bmj.com/site/about/guidelines.xhtml

data why the authors cannot share them. EPR data are however available from the corresponding author upon reasonable request.

List of abbreviations

ASA – American Society of Anesthesiologists Physical Classification

BMI – Body Mass Index

CCI – Charlson Comorbidity index

CI – Confidence interval

CT – Computed Tomography

EPR – Electronic patient records

Gallriks - The Swedish Registry of Gallstone Surgery and Endoscopic Retrograde Cholangiopancreatography

IQR – Interquartile range

NOM – Non-operative management

p – p-value

US - Ultrasound

Author contributions

EO and FL substantially contributed to the conception and design of the study. EO, SJ and CL acquired the data with the support of FL. EO and SJ performed analysis and interpretation of data. EO, SJ and FL drafted the article. All authors revised it critically for important intellectual content. All authors approved the final version to be published.

For peer review only - http://bmjopen.bmj.com/site/about/guidelines.xhtml

For peer review only

For peer review only - http://bmjopen.bmj.com/site/about/guidelines.xhtml

REFERENCES

1 Okamoto K, Suzuki K, Takada T, *et al.* Tokyo Guidelines 2018: flowchart for the management of acute cholecystitis. *J Hepato-Biliary-Pancreat Sci* 2018;**25**:55–72. doi:10.1002/jhbp.516

2 American College of Surgeons. COVID-19: Recommendations for Management of Elective Surgical Procedures. COVID-19 Recomm. Manag. Elect. Surg. Proced. 20200313.https://www.facs.org/for-medical-professionals/covid-19/clinical-guidance/elective-surgery/

3 Liberale G, Van Veer H, Lemaitre J, *et al.* Impact of the first wave of the SARS-CoV-2/Covid-19 pandemic on digestive surgical activities: a Belgian National Survey. *Acta Chir Belg* 2022;**122**:233–9. doi:10.1080/00015458.2021.1894735

4 Manzia TM, Angelico R, Parente A, *et al.* Global management of a common, underrated surgical task during the COVID-19 pandemic: Gallstone disease - An international survery. *Ann Med Surg* 2020;**57**:95–102. doi:10.1016/j.amsu.2020.07.021

5 Socialstyrelsen. Analys av första och andra covid-19-vågen – produktion, köer och väntetider i vården. 2021. https://www.socialstyrelsen.se/globalassets/sharepoint-dokument/artikelkatalog/ovrigt/2021-5-7371.pdf

6 Hale T, Angrist N, Goldszmidt R, *et al.* A global panel database of pandemic policies (Oxford COVID-19 Government Response Tracker). *Nat Hum Behav* 2021;**5**:529–38. doi:10.1038/s41562-021-01079-8

7 Francis N, Dort J, Cho E, *et al.* SAGES and EAES recommendations for minimally invasive surgery during COVID-19 pandemic. *Surg Endosc* 2020;**34**:2327–31. doi:10.1007/s00464-020-07565-w

8 Siriwardena AK, CHOLECOVID Collaborative, Spiers HVM, *et al.* Global overview of the management of acute cholecystitis during the COVID-19 pandemic (CHOLECOVID study). *BJS Open* 2022;**6**:zrac052. doi:10.1093/bjsopen/zrac052

9 Chrysos A, Amygdalos I, Nunes P, *et al.* The impact of the COVID-19 outbreak on emergency general surgery in the first German “hotspot region” Aachen-Heinsberg–A multicentre retrospective cohort study. *PLOS ONE* 2023;**18**:e0280867. doi:10.1371/journal.pone.0280867

10 Enochsson L, Thulin A, Österberg J, *et al.* The Swedish Registry of Gallstone Surgery and Endoscopic Retrograde Cholangiopancreatography (GallRiks): A Nationwide Registry for Quality Assurance of Gallstone Surgery. *JAMA Surg* 2013;**148**:471. doi:10.1001/jamasurg.2013.1221

11 Sandblom G, Österberg J, Rogmark P, *et al.* Impact of the COVID-19 pandemic on the management of gallstone surgery: A Swedish population-based study. *Scand J Surg* 2022;**111**:145749692110703. doi:10.1177/14574969211070389

12 Greenberg AL, Schwartz H, Collins CR, *et al.* Emergency general surgery utilization and disparities during COVID-19: an interrupted time-series analysis. *Trauma Surg Acute Care Open* 2021;**6**:e000679. doi:10.1136/tsaco-2021-000679

13 Balvardi S, Cipolla J, Touma N, *et al.* Impact of the Covid-19 pandemic on rates of emergency department utilization and hospital admission due to general surgery conditions. *Surg Endosc* 2022;**36**:6751–9. doi:10.1007/s00464-021-08956-3

14 Siegel R, Hohenstein S, Anders S, *et al.* Access to Surgery and Quality of Care for Acute Cholecystitis During the COVID-19 Pandemic in 2020 and 2021 - an Analysis of 12,545 Patients from a German-Wide Hospital Network. *J Gastrointest Surg Off J Soc Surg Aliment Tract* 2022;**26**:1462–71. doi:10.1007/s11605-022-05318-9

15 Yokoe M, Hata J, Takada T, *et al.* Tokyo Guidelines 2018: diagnostic criteria and

For peer review only

For peer review only - http://bmjopen.bmj.com/site/about/guidelines.xhtml

severity grading of acute cholecystitis (with videos). *J Hepato-Biliary-Pancreat Sci* 2018;**25**:41–54. doi:10.1002/jhbp.515

16 Benchimol EI, Smeeth L, Guttmann A, *et al.* The REporting of studies Conducted using Observational Routinely-collected health Data (RECORD) Statement. *PLOS Med* 2015;**12**:e1001885. doi:10.1371/journal.pmed.1001885

17 Osterman E, Helenius L, Larsson C, *et al.* Surgery for acute cholecystitis in severely comorbid patients: a population-based study on acute cholecystitis. *BMC Gastroenterol* 2022;**22**:371. doi:10.1186/s12876-022-02453-0

18 Public Health Agency of Sweden. Ny fas kräver nya insatser mot covid-19. Ny Fas Kräver Nya Insatser Mot Covid-19. 2020.https://www.folkhalsomyndigheten.se/nyheter-och-press/nyhetsarkiv/2020/mars/ny-fas-kraver-nya-insatser-mot-covid-19/ (accessed 31 May 2022).

19 Sax C, Eddelbuettel D. Seasonal Adjustment by X-13ARIMA-SEATS in R. *J Stat Softw* 2018;**87**. doi:10.18637/jss.v087.i11

20 Svenska Intensivvårdsregistret. 2019.http://portal.icuregswe.org/utdata/

21 ASA Physical Status Classification System. http://www.asahq.org/For-Members/Clinical-Information/ASA-Physical-Status-Classification-System.aspx (accessed 14 Aug 2013).

22 on behalf of the European Society of Radiology (ESR) and the European Society of Thoracic Imaging (ESTI), Revel M-P, Parkar AP, *et al.* COVID-19 patients and the radiology department – advice from the European Society of Radiology (ESR) and the European Society of Thoracic Imaging (ESTI). *Eur Radiol* 2020;**30**:4903–9. doi:10.1007/s00330-020-06865-y

23 Coleman JJ, Botkai A, Marson EJ, *et al.* Bringing into focus treatment limitation and DNACPR decisions: How COVID-19 has changed practice. *Resuscitation* 2020;**155**:172–9. doi:10.1016/j.resuscitation.2020.08.006

24 Briedé S, van Goor HMR, de Hond TAP, *et al.* Code status documentation at admission in COVID-19 patients: a descriptive cohort study. *BMJ Open* 2021;**11**:e050268. doi:10.1136/bmjopen-2021-050268

25 Brunn M, Kratz T, Padget M, *et al.* Why are there so many hospital beds in Germany? *Health Serv Manage Res* 2023;**36**:75–81. doi:10.1177/09514848221080691

26 Zangbar B, Rhee P, Pandit V, *et al.* Seasonal Variation in Emergency General Surgery. *Ann Surg* 2016;**263**:76–81. doi:10.1097/SLA.0000000000001238

27 Ljung R, Pihlström N, Sundström A. Risk of appendicitis after SARS-CoV-2 infection and SARS-CoV-2 vaccination. *Br J Surg* 2022;:znac127. doi:10.1093/bjs/znac127

For peer review only

For peer review only - http://bmjopen.bmj.com/site/about/guidelines.xhtml

FIGURE CAPTIONS

Figure 1: Number of patients admitted to the ICU with Covid-19 at the three hospitals and the changes in total cases of cholecystitis and the proportion treated with early surgery during the pandemic compared with 2017-2019.

For peer review only

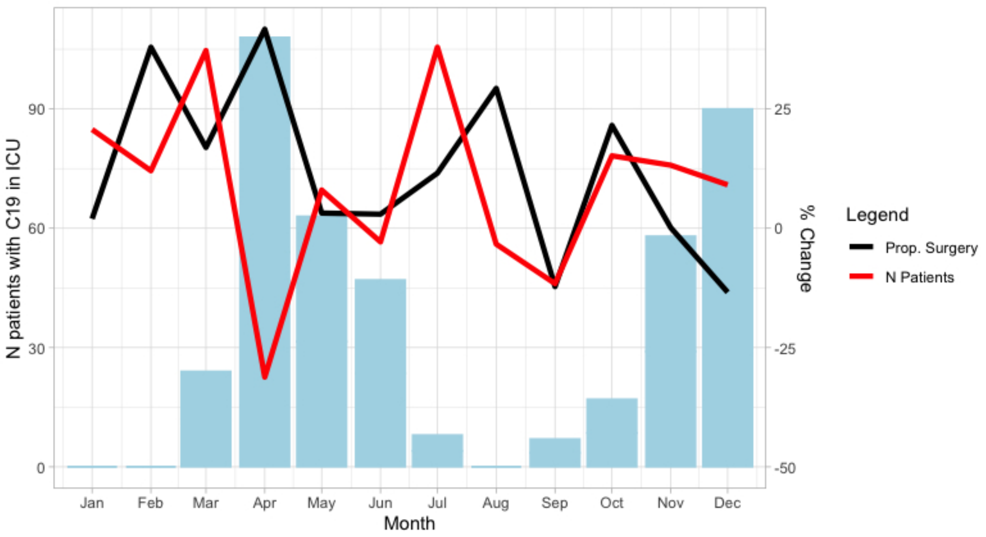

Figure 1: Number of patients admitted to the ICU with Covid-19 at the three hospitals and the changes in total cases of cholecystitis and the proportion treated with early surgery during the pandemic compared with 2017-2019.

275x149mm (72 x 72 DPI)

The RECORD statement – checklist of items, extended from the STROBE statement, that should be reported in observational studies using routinely collected health data.

	Item No.	STROBE items	Location in manuscript where items are reported	RECORD items	Location in manuscript where items are reported
**Title and abstract**					
	1	(a) Indicate the study's design with a commonly used term in the title or the abstract (b) Provide in the abstract an informative and balanced summary of what was done and what was found		RECORD 1.1: The type of data used should be specified in the title or abstract. When possible, the name of the databases used should be included.<br><br>RECORD 1.2: If applicable, the geographic region and timeframe within which the study took place should be reported in the title or abstract.<br><br>RECORD 1.3: If linkage between databases was conducted for the study, this should be clearly stated in the title or abstract.	1.1 Abstract: "Data was collected retrospectivel y from electronic patient records and the national registry for gallstone surgery"<br>1.2 "1,634 patients diagnosed with cholecystitis in two Swedish counties between 2017 and 2020 were included in the study."<br>1.3 See 1.1
**Introduction**					
Background rationale	2	Explain the scientific background and rationale for the investigation being reported			Background

For peer review only

Objectives	3	State specific objectives, including any prespecified hypotheses			Background second to last section
**Methods**					
Study Design	4	Present key elements of study design early in the paper			Method section 1
Setting	5	Describe the setting, locations, and relevant dates, including periods of recruitment, exposure, follow-up, and data collection			Method paragraph 2+3
Participants	6	*(a) Cohort study* - Give the eligibility criteria, and the sources and methods of selection of participants. Describe methods of follow-up<br>*Case-control study* - Give the eligibility criteria, and the sources and methods of case ascertainment and control selection. Give the rationale for the choice of cases and controls<br>*Cross-sectional study* - Give the eligibility criteria, and the sources and methods of selection of participants<br><br>*(b) Cohort study* - For matched studies, give matching criteria and number of exposed and unexposed<br>*Case-control study* - For matched studies, give matching criteria and the number of controls per case		RECORD 6.1: The methods of study population selection (such as codes or algorithms used to identify subjects) should be listed in detail. If this is not possible, an explanation should be provided.<br><br>RECORD 6.2: Any validation studies of the codes or algorithms used to select the population should be referenced. If validation was conducted for this study and not published elsewhere, detailed methods and results should be provided.<br><br>RECORD 6.3: If the study involved linkage of databases, consider use of a flow diagram or other graphical display to demonstrate the data linkage process, including the number of individuals with linked data at each stage.	6.1, 6.2, 6.3 Methods Reference to first paper describing the cohort.
Variables	7	Clearly define all outcomes, exposures, predictors, potential confounders, and effect		RECORD 7.1: A complete list of codes and algorithms used to classify exposures, outcomes, confounders, and effect modifiers should be provided. If	Method, Section 1 last paragraphs

For peer review only

		modifiers. Give diagnostic criteria, if applicable.		these cannot be reported, an explanation should be provided.	
Data sources/ measurement	8	For each variable of interest, give sources of data and details of methods of assessment (measurement).<br>Describe comparability of assessment methods if there is more than one group			Method, Reference to first paper with cohort
Bias	9	Describe any efforts to address potential sources of bias			Methods Reference to first paper with cohort
Study size	10	Explain how the study size was arrived at			Methods, All patients with diagnosis. Power analysis performed for first study. Power analysis reported in section Statistical analysis.
Quantitative variables	11	Explain how quantitative variables were handled in the analyses. If applicable, describe which groupings were chosen, and why			Methods, Section 1 paragraph 2 and reference to previous paper
Statistical methods	12	(a) Describe all statistical methods, including those used to control for confounding<br>(b) Describe any methods used to examine subgroups and interactions<br>(c) Explain how missing data were addressed<br>(d) *Cohort study* - If applicable, explain how loss to follow-up was addressed			a) Methods: Statistics, Paragraph 1<br>b) NA<br>c) Methods: Statistics, paragraph 1<br>d) Methods: Statistics,

For peer review only

		Case-control study - If applicable, explain how matching of cases and controls was addressed<br>*Cross-sectional study* - If applicable, describe analytical methods taking account of sampling strategy<br>(e) Describe any sensitivity analyses			paragraph 1<br>e) NA
Data access and cleaning methods		..		RECORD 12.1: Authors should describe the extent to which the investigators had access to the database population used to create the study population.<br><br>RECORD 12.2: Authors should provide information on the data cleaning methods used in the study.	12.2 Methods References first paper with cohort
Linkage		..		RECORD 12.3: State whether the study included person-level, institutional-level, or other data linkage across two or more databases. The methods of linkage and methods of linkage quality evaluation should be provided.	Methods, section 1, paragraph 2
**Results**					
Participants	13	(a) Report the numbers of individuals at each stage of the study (*e.g.*, numbers potentially eligible, examined for eligibility, confirmed eligible, included in the study, completing follow-up, and analysed)<br>(b) Give reasons for non-participation at each stage.<br>(c) Consider use of a flow diagram		RECORD 13.1: Describe in detail the selection of the persons included in the study (*i.e.,* study population selection) including filtering based on data quality, data availability and linkage. The selection of included persons can be described in the text and/or by means of the study flow diagram.	Methods section 1

For peer review only

Descriptive data	14	(a) Give characteristics of study participants (*e.g.*, demographic, clinical, social) and information on exposures and potential confounders<br>(b) Indicate the number of participants with missing data for each variable of interest<br>(c) *Cohort study* - summarise follow-up time (*e.g.*, average and total amount)			Results,<br>A+b) Table 1
Outcome data	15	*Cohort study* - Report numbers of outcome events or summary measures over time<br>*Case-control study* - Report numbers in each exposure category, or summary measures of exposure<br>*Cross-sectional study* - Report numbers of outcome events or summary measures			Results:<br>Temporal trends
Main results	16	(a) Give unadjusted estimates and, if applicable, confounder-adjusted estimates and their precision (e.g., 95% confidence interval). Make clear which confounders were adjusted for and why they were included<br>(b) Report category boundaries when continuous variables were categorized<br>(c) If relevant, consider translating estimates of relative risk into absolute risk for a meaningful time period			Results:<br>Delayed surgery, table 2<br>b) Done for period and otherwise NA<br>c) reported in "Delayed surgery and new gallstone complications" where applicable.
Other analyses	17	Report other analyses done—e.g., analyses of subgroups and			NA

For peer review only

		interactions, and sensitivity analyses			
**Discussion**					
Key results	18	Summarise key results with reference to study objectives			Discussion first few paragraphs
Limitations	19	Discuss limitations of the study, taking into account sources of potential bias or imprecision. Discuss both direction and magnitude of any potential bias		RECORD 19.1: Discuss the implications of using data that were not created or collected to answer the specific research question(s). Include discussion of misclassification bias, unmeasured confounding, missing data, and changing eligibility over time, as they pertain to the study being reported.	Discussion, Section Strengths and limitations
Interpretation	20	Give a cautious overall interpretation of results considering objectives, limitations, multiplicity of analyses, results from similar studies, and other relevant evidence			Discussion, first section paragraph 3-5
Generalisability	21	Discuss the generalisability (external validity) of the study results			Discussion, first section 5th paragraph
**Other Information**					
Funding	22	Give the source of funding and the role of the funders for the present study and, if applicable, for the original study on which the present article is based			Cover page
Accessibility of protocol, raw data, and programming code		..		RECORD 22.1: Authors should provide information on how to access any supplemental information such as the study protocol, raw data, or programming code.	Supplementary material and data availability

For peer review only

*Reference: Benchimol EI, Smeeth L, Guttmann A, Harron K, Moher D, Petersen I, Sørensen HT, von Elm E, Langan SM, the RECORD Working Committee. The REporting of studies Conducted using Observational Routinely-collected health Data (RECORD) Statement. *PLoS Medicine* 2015; in press.

*Checklist is protected under Creative Commons Attribution (CC BY) license.

For peer review only

Effect of the COVID-19 Pandemic on the Care for Acute Cholecystitis – a Swedish Multicenter Retrospective Cohort Study

Journal:	*BMJ Open*
Manuscript ID	bmjopen-2023-078407.R1
Article Type:	Original research
Date Submitted by the Author:	12-Oct-2023
Complete List of Authors:	Osterman, Erik; Uppsala University, CKF Gävleborg; Gävle sjukhus, Department of Surgery<br>Jakobsson, Sofia; Uppsala University, Department of Surgical Sciences<br>Larsson, Christina; Gävle sjukhus, Department of Surgery<br>Linder, Fredrik; Uppsala University Hospital, Department of Surgery; Uppsala University, Department of Surgical Sciences
**Primary Subject Heading**:	Surgery
Secondary Subject Heading:	Infectious diseases, Epidemiology
Keywords:	COVID-19, Observational Study, Retrospective Studies, SURGERY

I, the Submitting Author has the right to grant and does grant on behalf of all authors of the Work (as defined in the below author licence), an exclusive licence and/or a non-exclusive licence for contributions from authors who are: i) UK Crown employees; ii) where BMJ has agreed a CC-BY licence shall apply, and/or iii) in accordance with the terms applicable for US Federal Government officers or employees acting as part of their official duties; on a worldwide, perpetual, irrevocable, royalty-free basis to BMJ Publishing Group Ltd ("BMJ") its licensees and where the relevant Journal is co-owned by BMJ to the co-owners of the Journal, to publish the Work in this journal and any other BMJ products and to exploit all rights, as set out in our licence.

The Submitting Author accepts and understands that any supply made under these terms is made by BMJ to the Submitting Author unless you are acting as an employee on behalf of your employer or a postgraduate student of an affiliated institution which is paying any applicable article publishing charge ("APC") for Open Access articles. Where the Submitting Author wishes to make the Work available on an Open Access basis (and intends to pay the relevant APC), the terms of reuse of such Open Access shall be governed by a Creative Commons licence – details of these licences and which Creative Commons licence will apply to this Work are set out in our licence referred to above.

Other than as permitted in any relevant BMJ Author's Self Archiving Policies, I confirm this Work has not been accepted for publication elsewhere, is not being considered for publication elsewhere and does not duplicate material already published. I confirm all authors consent to publication of this Work and authorise the granting of this licence.

For peer review only

Effect of the COVID-19 Pandemic on the Care for Acute Cholecystitis – a Swedish Multicenter Retrospective Cohort Study

Authors

Erik Osterman MD PhD [1,4] *† (ORCID 0000-0003-1621-7872), Sofia Jakobsson[3] † (ORCID 0009-0003-2648-6213), Christina Larsson MD[1], Fredrik Linder MD PhD [2,3] (ORCID 0000-0001-8125-4536)

Affiliations

1 Department of Surgery, Gävle Hospital, Gävleborg Region, Sweden

2 Department of Surgery, Uppsala University Hospital, Uppsala Region, Sweden

3 Department of Surgical Sciences, Uppsala University, Sweden

4 Centre for Clinical Research Gävleborg, Uppsala University, Sweden

*Corresponding author

† Contributed equally to this paper

Corresponding author: Erik Osterman, Department of Surgery, Gävle Hospital, Region Gävleborg 80187 Sweden

erik.osterman@igp.uu.se erik.osterman@regiongavleborg.se

Cell: +46735838735. Work: +4626157685

Manuscript type: Original Article

Word count: Main text 3 117 words + Tables 505 words + Statements 309 words

Abstract: 297

For peer review only

ABSTRACT

Objectives: The present study aimed to investigate if and how the panorama of acute cholecystitis changed in 2020 in Sweden. Seven aspects were identified, the incidence of cholecystitis, the Tokyo grade, the timing of diagnosis and treatment, the proportion treated with early surgery, the proportion of patients treated with delayed surgery, and new complications from gallstones.

Design: Retrospective multicenter cohort study

Setting: 3 hospitals in Sweden, covering 675,000 inhabitants

Participants: 1,634 patients with cholecystitis

Outcomes: The incidence, treatment choice and diagnostic and treatment delay were investigated by comparing pre-pandemic and pandemic patients.

Results: Patients diagnosed with cholecystitis during the pandemic were more comorbid (ASA2-5 86% vs 81%, p 0.01) and more often had a diagnostic computed tomography (67% vs 59% p 0.01). There were variations in the number of patients corresponding with the pandemic waves, but there was no overall increase in the number of patients with cholecystitis (78 vs 76 cases/100,000 inhabitants, p 0.7) or the proportion of patients treated with surgery during the pandemic (50% vs 50%, p 0.4). There was no increase in time to admission from symptoms (both median 1 day, p 0.7), or surgery from admission (both median 1 day, p 0.9). The proportion of grade 2-3 cholecystitis was not higher during the pandemic (46% vs 44% p 0.9). The median time to elective surgery increased (184 days vs 130 days, p 0.04), but there was no increase in new gallstone complications (35% vs 39% p 0.3).

Conclusion: Emergency surgery for cholecystitis was not impacted by the pandemic in Sweden. Patients were more comorbid but did not have more severe cholecystitis nor was there a delay in seeking care. Fewer patients non-operatively managed had elective surgery

For peer review only

within 6 months of their initial diagnosis but there was no corresponding increase in gallstone complications.

For peer review only

For peer review only - http://bmjopen.bmj.com/site/about/guidelines.xhtml

Strengths and limitations of this study

- Covers all patients seeking hospital care for cholecystitis in a developed country with few restrictions
- Utilizes both electronic patient records and prospectively collected registry data
- Any patient solely cared for in primary care not included
- Only includes 2020 as a pandemic year
- Mainly Retrospective study

BACKGROUND

Early cholecystectomy is the recommended treatment for acute cholecystitis. Non-operatively managed (NOM) patients may be considered for surgery later to avoid new complications from cholecystolithiasis.[1] During the coronavirus disease 2019 (COVID-19) pandemic, some elective surgical procedures were deferred to handle the effects of the pandemic.[2] The pandemic affected uncomplicated cholecystectomies more than other procedures[3,4], and the number of elective cholecystectomies in Sweden decreased by 30% while emergency procedures only decreased by 1%.[5] In 2020 the pandemic hit Sweden in two waves, the first starting in March and the second in October.[5] Sweden had less strict policies to stop the spread of the virus than many other countries.[6]

Previous studies

Increased severity of cholecystitis and fewer patients being treated with early cholecystectomy for cholecystitis have been seen during the COVID-19 pandemic.[7,8] The Swedish Registry of Gallstone Surgery and Endoscopic Retrograde

Cholangiopancreatography (Gallriks)[9] reported fewer elective procedures while emergency procedures increased, but no data were available on non-operative management.[10] The incidence of cholecystitis during the pandemic has not been completely investigated, cholecystitis decreased in the US[11] while it remained the same in Canada,[12] increased in Germany[8] and the CHOLECOVID study[7] showed a decrease in mild cholecystitis but not moderate and severe cholecystitis. Emergency department visits fell in many places [5,11,12] which could indicate that patients waited at home and then presented after 5-7 days when conservative treatment is recommended explaining falling emergency surgery numbers. However, a German study investigated the proportion of non-surgically treated patients and found that non-operative management did not increase during the pandemic.[13]

Aims and hypothesis

The present study aimed to investigate if and how the panorama of acute cholecystitis changed during 2020 at three Swedish centers in two counties. Seven aspects were identified: the incidence of cholecystitis, the Tokyo grade,[14] the timing of diagnosis and treatment, the proportion treated with early surgery, the proportion of patients treated with delayed surgery, and new complications from gallstones. The hypothesis was that the incidence of cholecystitis and the proportion treated with early surgery did not change while the grade increased, and patients were diagnosed later. We also hypothesized that fewer non-operatively treated patients had surgery within 6 months of discharge and more patients had new gallstone complications if diagnosed in 2020.

For peer review only - http://bmjopen.bmj.com/site/about/guidelines.xhtml

METHOD

The Swedish Ethical Review Authority approved the study, number 2021-00862. Patients with data from Gallriks had consented to be included in the registry for research purposes, for patients not included in Gallriks or treated NOM the Swedish Ethical Review Authority waived the requirement of consent since the study was retrospective and without intervention or risk to the patients. The manuscript was prepared following the Reporting of studies Conducted using Observational Routinely-collected Data (RECORD) checklist.[15] Patients or the public were not involved in the design, conduct, reporting, or dissemination plans of our research.

Patients with a diagnosis of acute cholecystitis between 2017-2020 were identified by the administrative data offices of Region Gävleborg and Region Uppsala. The data was then gathered from electronic patient records (EPR) and Gallriks as described previously.[16] The chart review started in mid-2021 and finished in early 2022. The possibility of chart review for the study staff determined the choice of regions.

The outcomes were: Incidence of cholecystitis (number of cases/100,000 inhabitants), grade (G1: mild or G2-3: moderate/severe),[14] the time to first contact with the emergency department and diagnosis from symptoms in days, time to surgery, treatment choice (surgery or NOM), time to delayed surgery, the proportion NOM patients who underwent surgery within 6 months and new gallstone complication after discharge. Exposure was a diagnosis of acute cholecystitis during the pandemic (after March 13 2020).[17] Patients were stratified by diagnosis time, pre-pandemic or pandemic to analyze outcomes and demographics. To assess delays in delayed surgery all patients diagnosed during 2020 were analyzed as the exposed group. The number of inhabitants over 18 years of age in the two regions was obtained from

For peer review only

Statistics Sweden and was transformed to person-years of exposure. The results were then checked against the national patient registry to further investigate the incidence of cholecystitis.

Statistical analysis

Differences between groups were tested with the χ^2 test for categorical variables and the Wilcoxon rank sum test for discrete variables. Post-hoc testing with Holm correction for multiple testing was used for variables significant with more than two levels. The incidence was analyzed in two ways, first calculated from the monthly rate and adjusted for the population size of the counties, and second by also adjusting for seasonality with the X11 procedure.[18] Logistic regression was used to analyze the proportion of NOM patients undergoing elective surgery within 6 months of their cholecystitis adjusting for comorbidities. A Cox proportional hazards model was used to investigate the time to new gallstone complications censoring for death and surgery. The number of patients each month and the proportion having surgery compared to the previous period were plotted. The monthly number of ICU admissions for COVID-19 was overlaid to convey the waves of the pandemic, the data came from the Swedish Intensive Care Registry's open reports.[19] Data was not imputed, and complete case analysis was performed where applicable. Power analysis for an effect size of 0.3 with 95% power and α 0.05, for the Wilcoxon rank sum test and χ^2 test with 4 degrees of freedom gave a required sample of at least 854 and 207 patients which determined the number of control years to include in the study. Logistic regression required a sample size of 636 to detect a 25% decrease in elective surgery within 6 months at 95% power and α 0.05. The Cox proportional hazards regression required a sample size of 954 to detect an increase of 50% (HR 1.5) in new complications at 95% power and α 0.05. Statistics were calculated with R version 4.2.2 (Vienna, Austria).

For peer review only - http://bmjopen.bmj.com/site/about/guidelines.xhtml

RESULTS

In total, 1,634 patients were included in the study, 1,286 patients were diagnosed before the pandemic and 348 after the start of the pandemic. The incidence of first-time cholecystitis did not change during the pandemic (76/100,000 cases/person-years pre-pandemic vs 81/100,000 cases/person-years, p 0.3, χ^2 test, data in table 1). Adjusting for seasonality did not change the result. The national patient registry did not show a significant change in the incidence of cholecystitis between the years 2017-2019 and 2020-2021 either.

There was no difference in sex, age, or BMI between the two periods, table 2. During the pandemic, there was a 13% increase in patients classified as 2 in the American Society of Anesthesiologists physical classification system (ASA).[20] There was a corresponding 22% decrease in otherwise healthy patients (post-hoc test ASA1 p 0.23 and ASA2 p 0.08). More patients had a computed tomography (CT) as a diagnostic modality during the pandemic (59% pre-pandemic vs 67%, post-hoc test p 0.007). Five patients had concurrent COVID-19.

Table 1: Incidence of new cholecystitis 2017-2020, excluding recurrences during that period.

	Period	Person-years	Cases	Cases/100,000	p
Unadjusted	***Pre-pandemic***	1,685,729	1286	76	0.3
	Pandemic *(From 2020-03-13)*	429,600	348	81	
Seasonally adjusted	***Pre-pandemic***	1,713,119	1297	76	0.7
	Pandemic *(From 2020-04-01)*	429,600	313	78	

P value calculated with χ^2 test, only persons ≥18 years old used for person-years.

Unchanged grade and admission times

There was no difference in the proportion of grade 1 versus grade 2-3 cholecystitis between the two periods, table 2. No difference in leukocyte counts and C-reactive protein at

For peer review only

admission was noted suggesting that patients were not admitted later in the disease course (median leukocyte count 12.3x10^9/l pre-pandemic vs 12.3x10^9/l, p 0.53, median CRP 74 mg/l pre-pandemic vs 83 mg/l, p 0.62, Wilcoxon rank sum test). This was confirmed by a lack of differences in time to first contact with the emergency department and admission after symptoms. No difference in time to surgery from admission was noted either suggesting that patients treated with surgery had the same priority as before the pandemic. There was no difference in the length of stay for patients treated with surgery (median 3 days, p 0.75) or without surgery (median 4 days, p 0.95).

Treatment choice

There was no difference in the proportion of patients treated with early surgery during the pandemic (44% pre-pandemic vs 46%, p 0.41), table 2. There was no difference in the proportion of laparoscopic (38% pre-pandemic vs 38%), open (4% pre-pandemic vs 5%) or surgeries converted to open (2% pre-pandemic vs 3%) between the two periods (p 0.14). For patients NOM there was no difference in the proportion receiving drains or the plan for follow-up, 24% of patients were planned for delayed surgery either at discharge or after a visit to the out-patient clinic (p 0.24). Surprisingly there was no difference in the proportion of patients referred to the outpatient clinic during the pandemic with 38% of NOM patients being referred and about 40% of those being planned for surgery during both periods (p 0.45).

For peer review only - http://bmjopen.bmj.com/site/about/guidelines.xhtml

Table 2: Demographics, treatment and outcomes for patients before and during the COVID-19 pandemic.

	Total	Pre-pandemic	Pandemic	p
**Sex**				
Male	857 (52%)	666 (52%)	191 (55%)	0.33
Female	777 (48%)	620 (48%)	157 (45%)	
**Age**				
Median (IQR)	67.2 (51.1 - 77.0)	67.2 (50.7 - 76.8)	67.2 (53.9 - 77.5)	0.27
**BMI**				
Median (IQR)	27.8 (24.7 - 31.4)	27.8 (24.7 - 31.3)	27.9 (24.7 - 31.5)	0.98
Missing	117 (7.2%)	93 (7.2%)	24 (6.9%)	
**ASA**				
1	300 (18%)	250 (19%)	50 (14%)	0.013
2	731 (45%)	554 (43%)	177 (51%)	
3	515 (32%)	405 (31%)	110 (32%)	
4	87 (5%)	76 (6%)	11 (3%)	
5	1 (0%)	1 (0%)	0 (0%)	
**CCI**				
Median (IQR)	3.0 (1.0 - 5.0)	3.0 (1.0 - 5.0)	3.0 (1.0 - 5.0)	0.43
**Previous stone**				
No	1,176 (72%)	919 (71%)	257 (74%)	0.42
Yes	458 (28%)	367 (29%)	91 (26%)	
**Imaging**				
No radiology	59 (4%)	50 (4%)	9 (3%)	0.01
US	579 (35%)	475 (37%)	104 (30%)	
CT	570 (35%)	424 (33%)	146 (42%)	
CT+US	424 (26%)	336 (26%)	88 (25%)	
Other+MRCP	2 (0%)	1 (0%)	1 (0%)	
**Cholecystitis Grade**				
G1	798 (49%)	627 (49%)	171 (49%)	0.85
G2-3	729 (45%)	569 (44%)	160 (46%)	
Missing	107 (7%)	90 (7%)	17 (5%)	
**Treatment**				
Conservative	815 (50%)	642 (50%)	173 (50%)	0.41
Surgery	725 (44%)	565 (44%)	160 (46%)	
Drain	94 (6%)	79 (6%)	15 (4%)	
**Time to first contact from symptoms**				
Median (IQR)	1.0 (0.0 - 3.0)	1.0 (0.0 - 3.0)	1.0 (0.0 - 3.0)	0.78
Missing	57 (3.5%)	45 (3.5%)	12 (3.4%)	
**Time to admission from symptoms**				
Median (IQR)	2.0 (1.0 - 3.0)	2.0 (1.0 - 3.0)	2.0 (1.0 - 3.0)	0.7
Missing	107 (6.5%)	89 (6.9%)	18 (5.2%)	
**Time to surgery from admission**				
Median (IQR)	1.0 (1.0 - 2.0)	1.0 (1.0 - 2.0)	1.0 (1.0 - 2.0)	0.86
**Length of stay (days)**				
Median (IQR)	3.0 (2.0 - 6.0)	3.0 (2.0 - 6.0)	3.0 (2.0 - 5.0)	0.79
Missing	52 (3.2%)	45 (3.5%)	7 (2.0%)	
**Plan for follow-up**				
No	829 (73%)	657 (72%)	172 (76%)	0.24
Surgery	304 (27%)	251 (28%)	53 (24%)	
**Readmitted within 30 days**				
No	1,445 (88%)	1,138 (88%)	307 (88%)	0.92
Yes	189 (12%)	148 (12%)	41 (12%)	

Fisher exact test used for categorical variables, Wilcoxon rank sum test for discrete variables.
ASA: American Society of Anesthesiologists Performance Status Classification, BMI: Body Mass Index, CCI: Charlson Comorbidity Index. IQR: Interquartile range. US: Ultrasound

For peer review only

For peer review only - http://bmjopen.bmj.com/site/about/guidelines.xhtml

Temporal trends

The number of cases and proportion treated with early surgery was calculated and compared with the three previous years to investigate if the pandemic changed the number of patients and the treatment choice month by month, figure 1. During the first three months of the year, there were approximately 20% more patients than the previous years and the proportion treated with early surgery was higher, in April as the pandemic hit the total number of cases went down and a larger proportion of patients had surgery. During the second wave starting in October, the proportion treated with early surgery fell while cases remained comparable to 2017-2019 numbers. Changes were not statistically significant, and the figure is meant to illustrate the changes that were seen during the pandemic.

Delayed surgery and new gallstone complications

The Tokyo guidelines consider delayed surgery to be surgery performed at least 42 days after acute cholecystitis.[1] The median time to surgery in the two regions in 2017-2019 was 130 days (interquartile range, IQR 76, 214) while it was 184 days (IQR 78,341) in 2020 (p 0.04 Wilcoxon rank sum test). In 2017-2019 20% of NOM patients had surgery within 6 months while in 2020 only 11% had surgery within this time (p 0.003, χ^2 test). The only patient variable differing between the periods was ASA classification, and since this might affect the proportion treated with early surgery it was included in the logistic regression, table 3. Adjusted odds ratio (OR) for surgery within 6 months in NOM patients was 0.47 (95%CI 0.29-0.74, p 0.002). This is in line with the longer median time to surgery seen in the unadjusted analysis.

The risk of new gallstone complications was not impacted by this as 39% of patients NOM in 2017-2019 and 35% in 2020 had recurrent gallstone disease (p 0.3, χ^2test). There was no change in the pattern of new gallstone complications with new cholecystitis in 140 of 721

For peer review only - http://bmjopen.bmj.com/site/about/guidelines.xhtml

patients pre-pandemic (19%) and 33 of 188 (18%) patients during the pandemic. Cholangitis was the first complication in 14 patients (2%) pre-pandemic and 6 patients (3%) during the pandemic. Pancreatitis was the first complication in 11 patients (2%) pre-pandemic and 5 patients (3%) during the pandemic. The median time to a new gallstone complication was 87 days (IQR 29-249) pre-pandemic and 76 days (IQR 23-194) during the pandemic (n.s.). Accounting for the follow-up with a Cox proportional hazards model confirmed that there was no difference, HR 0.91 (95%CI 0.67-1.22, p 0.5).

Table 3: Logistic regression for delayed surgery within 6 months

Characteristic	OR[1]	95% CI[1]	p-value
**Year**			
**2017-2019**	—	—	
**2020**	0.47	0.29, 0.74	0.002
**ASA**			
**1**	—	—	
**2**	0.72	0.45, 1.17	0.2
**3**	0.22	0.13, 0.38	<0.001
**4**	0.05	0.01, 0.16	<0.001

[1] OR = Odds Ratio, CI = Confidence Interval

For peer review only

For peer review only - http://bmjopen.bmj.com/site/about/guidelines.xhtml

DISCUSSION

During the first month of the pandemic, there were fewer patients seeking hospital care for cholecystitis, which was compensated by more patients later in the year. Overall, there was no difference in the incidence of first-time cholecystitis during the pandemic. The proportion of patients who had early surgery remained the same over time, but some months had a higher proportion of early surgery than previous years, despite patients diagnosed during the pandemic being more comorbid. This might have been a result of knowing that resources for delayed surgery were scarce, as shown in this paper and previous research from Gallriks. [10] The proportion of patients having early surgery was low in both periods, but not lower than previous Swedish evaluations. [21] The practice in Sweden has been restrictive on early surgery, limiting this to mildly comorbid patients and those presenting within 5 days of symptoms. Due to concerns for aerosolized virus in asymptomatic patients open surgery was recommended initially,[22] however no difference in the rate of open surgery was seen between the two periods.

The differences in ASA classification between the periods could be from otherwise healthy patients staying at home, thinking they had COVID-19. Testing capacity was limited at the beginning of 2020 thus abdominal pain and fever might have been considered COVID-19 instead of cholecystitis. Chest CT was sometimes performed instead of a nasopharyngeal swab which could explain the higher proportion of CT use.[23] Limited testing may also explain the low number of patients with concurrent COVID-19 infection. Another reason might be that ultrasound (US) was avoided to prevent close contact and transmission of infection. A third reason for the increase in CT use could be the limited availability of hospital beds, which could have led to CT being used as a tool to decide which patients needed to be admitted instead of waiting for further tests and US.

For peer review only - http://bmjopen.bmj.com/site/about/guidelines.xhtml

Comorbid patients might be more likely to seek out healthcare, especially if they fear that they have contracted a potentially deadly infection. This is similar to what the CHOLECOVID collaboration found, that patients were sicker during the pandemic.[7] Another possibility is that the documentation of comorbidities got better during the pandemic. However, the CCI was unchanged, suggesting that it was diseases not included in the CCI that led to ASA1 patients being classified as ASA2, e.g. hypertension or smoking. Two studies have compared the documentation of code status before and during the pandemic finding that it improved in a UK hospital from a low level and found no change in a Dutch hospital which was already at a high level of documentation.[24,25] However, the documentation of comorbidities has not been investigated in any study.

There was no increase in the grade of cholecystitis and the patients who did seek care did not do so later and were admitted and operated upon in a similar time frame as the previous three years, which was not investigated by CHOLECOVID or the national Swedish study.[7,10] There were no other indications that patients presented later, e.g. the leukocyte count or CRP. Emergency surgical care for patients with cholecystitis does not seem to have been affected by the pandemic in Sweden. If this is the result of universal healthcare in contrast with the United States or from more relaxed policies in contrast with Germany and Canada is not entirely clear.[11,12] The incidence and proportion of operatively managed patients did not decrease in Canada, which has universal healthcare but had tougher policies initially. In the United States there was a demographic shift towards younger and privately insured patients compared to before the pandemic.[11] In Germany, with the strictest policies of all, the incidence of cholecystitis rose.[8] However, it is not entirely comparable to Sweden since it runs an insurance-based healthcare system with an abundance of hospital beds.[26]

For peer review only - http://bmjopen.bmj.com/site/about/guidelines.xhtml

The follow-up and subsequent delayed surgery for NOM patients were further delayed by the pandemic. This is in line with guidelines at the time prioritizing emergency surgery and malignancy, and previous research findings.[2,10] The median wait time for delayed cholecystectomy was too long before the pandemic and the limited resources of the pandemic increased it by almost two months. Despite this, there was no increase in recurrent gallstone complications when adjusting for follow-up of patients diagnosed during the pandemic. Previous research in the same population highlighted that recurrent gallstone disease is most common in the first months after discharge and common in NOM patients.[16] Thus, delayed surgery did not stop them previously, and delaying it further did not change this.

Strengths and limitations

One of the main strengths of the study is the inclusion of all patients, not only those undergoing surgery. However, patients cared for by general practitioners would be missing from the analysis. Based on our clinical experience, most of these patients were previously referred to the hospital if they had a fever and abdominal pain making this less of an issue. However, they may not have been referred during the pandemic if they were otherwise healthy or thought to have abdominal symptoms from COVID-19.

This study included patients throughout the entirety of 2020 compared with CHOLECOVID which only included patients from the first two months of the pandemic. The risk of selection bias is lower than in a prospective study though with the drawbacks of a retrospective study where inclusion was dependent on correct classification in the EPR. Standardization of data collection was attempted and by utilizing EPR data recall bias is limited. However, some notes lacked in detail. The data is likely representative of countries with similar healthcare

For peer review only - http://bmjopen.bmj.com/site/about/guidelines.xhtml

systems and pandemic responses. Sensitivity analysis gave a required effect size of 0.22 for the Wilcoxon Ranked sum test to detect a difference at 95% power and α 0.05 and 0.1 for the X^2 test to detect a difference at 4 degrees of freedom. The logistic regression was sufficiently powered, while the Cox proportional hazards regression was powered to detect an increase of 50% in complications, thus smaller differences are not ruled out.

The study only investigated the treatment for the first cholecystitis in the period 2017-2020 and subsequent events were classified as new gallstone complications. Patients with their first cholecystitis at the end of 2019 would also be impacted by the pandemic and a reduced capacity for elective surgery in 2020. The subsequent waves in 2021 were not included in this study thus the study is limited to the initial response and changed policies. The pandemic group does not include the first two months of the year, where fewer patients have cholecystitis due to seasonal variation. The comparisons with previous years were performed on a month-to-month basis and the incidence was adjusted for season to account for this.[27]

Future perspectives

This study investigated the first cholecystitis in patients during the period 2017-2020 and did not find a change in the incidence. While there was no increased incidence of cholecystitis in the present study, other emergency general surgery diseases were affected by the pandemic, e.g. the incidence of appendicitis increased according to Swedish studies, potentially as a direct effect of COVID-19 infection.[28] The national patient registry which contains data on diagnoses and surgical treatment could allow for the evaluation of a national material and cover other EGS diseases besides cholecystitis. It would be especially interesting to investigate emergency general surgery diseases that are usually treated without surgery or

For peer review only

antibiotics, e.g. diverticulitis, to assess if patients stayed away from the hospitals during the pandemic.

Conclusion

At our centers, emergency surgery for cholecystitis was not impacted by the pandemic. Patients were more comorbid but did not have a more severe grade of cholecystitis nor did they seek care later. Fewer patients NOM had elective surgery within 6 months of their initial diagnosis but there was no corresponding increase in gallstone complications.

STATEMENTS, DISCLOSURES, ETC.

Acknowledgements

We want to thank the EPR and analysis departments at Region Gävleborg and Region Uppsala for their help with identifying the population from ICD codes. We also want the thank the surgeons and administrators reporting to Gallriks for making the study possible. Thanks to Anders Blomberg and Jennie Wickenberg for discussions regarding the design of the study. Thanks to Louise Helenius and Tamali Majumder for helping with the collection of data.

Ethical approval

The Swedish Ethical Review Authority approved the study, number 2021-00862. Patients with data from Gallriks had consented to be included in the registry for research purposes, for patients not included in Gallriks or treated NOM the Swedish Ethical Review Authority waived the requirement of consent since the study was retrospective and without intervention or risk to the patients.

For peer

For peer review only - http://bmjopen.bmj.com/site/about/guidelines.xhtml

Disclosure, Competing interests

No conflicts of interest.

Patient and Public Involvement

Patients or the public were not involved in the design, conduct, reporting, or dissemination plans of our research.

Funding

The research was funded by the Centre for Research and Development, Gävleborg Region grant numbers CFUG-965514, CFUG-965517. EO, LH, CL, AB, JW are funded by Region Gävleborg, no grant number. FL was financed by grants from the Swedish state under the agreement between the Swedish Government and the Uppsala County Council (ALF), no grant number. Open access funding from Uppsala University, no grant number.

Availability of data and materials

The data are not publicly available due to information that could compromise the privacy of research participants. The registry data that support the findings of this study are available from Gallriks (https://www.ucr.uu.se/gallriks/). Restrictions apply to the availability of these data why the authors cannot share them. EPR data are however available from the corresponding author upon reasonable request.

List of abbreviations

ASA – American Society of Anesthesiologists Physical Classification

BMI – Body Mass Index

For peer review only - http://bmjopen.bmj.com/site/about/guidelines.xhtml

CCI – Charlson Comorbidity index

CI – Confidence interval

CT – Computed Tomography

EPR – Electronic patient records

Gallriks - The Swedish Registry of Gallstone Surgery and Endoscopic Retrograde Cholangiopancreatography

IQR – Interquartile range

NOM – Non-operative management

p – p-value

US - Ultrasound

Author contributions

EO and FL substantially contributed to the conception and design of the study. EO, SJ and CL acquired the data with the support of FL. EO and SJ performed analysis and interpretation of data. EO, SJ and FL drafted the article. All authors revised it critically for important intellectual content. All authors approved the final version to be published.

For peer review only

REFERENCES

1 Okamoto K, Suzuki K, Takada T, *et al.* Tokyo Guidelines 2018: flowchart for the management of acute cholecystitis. *J Hepato-Biliary-Pancreat Sci* 2018;**25**:55–72. doi:10.1002/jhbp.516

2 American College of Surgeons. COVID-19: Recommendations for Management of Elective Surgical Procedures. COVID-19 Recomm. Manag. Elect. Surg. Proced. 20200313.https://www.facs.org/for-medical-professionals/covid-19/clinical-guidance/elective-surgery/

3 Liberale G, Van Veer H, Lemaitre J, *et al.* Impact of the first wave of the SARS-CoV-2/Covid-19 pandemic on digestive surgical activities: a Belgian National Survey. *Acta Chir Belg* 2022;**122**:233–9. doi:10.1080/00015458.2021.1894735

4 Manzia TM, Angelico R, Parente A, *et al.* Global management of a common, underrated surgical task during the COVID-19 pandemic: Gallstone disease - An international survery. *Ann Med Surg* 2020;**57**:95–102. doi:10.1016/j.amsu.2020.07.021

5 Socialstyrelsen. Analys av första och andra covid-19-vågen – produktion, köer och väntetider i vården. 2021. https://www.socialstyrelsen.se/globalassets/sharepoint-dokument/artikelkatalog/ovrigt/2021-5-7371.pdf

6 Hale T, Angrist N, Goldszmidt R, *et al.* A global panel database of pandemic policies (Oxford COVID-19 Government Response Tracker). *Nat Hum Behav* 2021;**5**:529–38. doi:10.1038/s41562-021-01079-8

7 Siriwardena AK, CHOLECOVID Collaborative, Spiers HVM, *et al.* Global overview of the management of acute cholecystitis during the COVID-19 pandemic (CHOLECOVID study). *BJS Open* 2022;**6**:zrac052. doi:10.1093/bjsopen/zrac052

8 Chrysos A, Amygdalos I, Nunes P, *et al.* The impact of the COVID-19 outbreak on emergency general surgery in the first German “hotspot region” Aachen-Heinsberg–A multicentre retrospective cohort study. *PLOS ONE* 2023;**18**:e0280867. doi:10.1371/journal.pone.0280867

9 Enochsson L, Thulin A, Österberg J, *et al.* The Swedish Registry of Gallstone Surgery and Endoscopic Retrograde Cholangiopancreatography (GallRiks): A Nationwide Registry for Quality Assurance of Gallstone Surgery. *JAMA Surg* 2013;**148**:471. doi:10.1001/jamasurg.2013.1221

10 Sandblom G, Österberg J, Rogmark P, *et al.* Impact of the COVID-19 pandemic on the management of gallstone surgery: A Swedish population-based study. *Scand J Surg* 2022;**111**:145749692110703. doi:10.1177/14574969211070389

11 Greenberg AL, Schwartz H, Collins CR, *et al.* Emergency general surgery utilization and disparities during COVID-19: an interrupted time-series analysis. *Trauma Surg Acute Care Open* 2021;**6**:e000679. doi:10.1136/tsaco-2021-000679

12 Balvardi S, Cipolla J, Touma N, *et al.* Impact of the Covid-19 pandemic on rates of emergency department utilization and hospital admission due to general surgery conditions. *Surg Endosc* 2022;**36**:6751–9. doi:10.1007/s00464-021-08956-3

13 Siegel R, Hohenstein S, Anders S, *et al.* Access to Surgery and Quality of Care for Acute Cholecystitis During the COVID-19 Pandemic in 2020 and 2021 - an Analysis of 12,545 Patients from a German-Wide Hospital Network. *J Gastrointest Surg Off J Soc Surg Aliment Tract* 2022;**26**:1462–71. doi:10.1007/s11605-022-05318-9

14 Yokoe M, Hata J, Takada T, *et al.* Tokyo Guidelines 2018: diagnostic criteria and severity grading of acute cholecystitis (with videos). *J Hepato-Biliary-Pancreat Sci* 2018;**25**:41–54. doi:10.1002/jhbp.515

15 Benchimol EI, Smeeth L, Guttmann A, *et al.* The REporting of studies Conducted

For peer review only - http://bmjopen.bmj.com/site/about/guidelines.xhtml

using Observational Routinely-collected health Data (RECORD) Statement. *PLOS Med* 2015;**12**:e1001885. doi:10.1371/journal.pmed.1001885

16 Osterman E, Helenius L, Larsson C, *et al.* Surgery for acute cholecystitis in severely comorbid patients: a population-based study on acute cholecystitis. *BMC Gastroenterol* 2022;**22**:371. doi:10.1186/s12876-022-02453-0

17 Public Health Agency of Sweden. Ny fas kräver nya insatser mot covid-19. Ny Fas Kräver Nya Insatser Mot Covid-19. 2020.https://www.folkhalsomyndigheten.se/nyheter-och-press/nyhetsarkiv/2020/mars/ny-fas-kraver-nya-insatser-mot-covid-19/ (accessed 31 May 2022).

18 Sax C, Eddelbuettel D. Seasonal Adjustment by X-13ARIMA-SEATS in R. *J Stat Softw* 2018;**87**. doi:10.18637/jss.v087.i11

19 Svenska Intensivvårdsregistret. 2019.http://portal.icuregswe.org/utdata/

20 ASA Physical Status Classification System. http://www.asahq.org/For-Members/Clinical-Information/ASA-Physical-Status-Classification-System.aspx (accessed 14 Aug 2013).

21 Pieniowski E, Popowicz A, Lundell L, *et al.* Early versus Delayed Surgery for Acute Cholecystitis as an Applied Treatment Strategy When Assessed in a Population-Based Cohort. *Dig Surg* 2014;**31**:169–76. doi:10.1159/000363659

22 Francis N, Dort J, Cho E, *et al.* SAGES and EAES recommendations for minimally invasive surgery during COVID-19 pandemic. *Surg Endosc* 2020;**34**:2327–31. doi:10.1007/s00464-020-07565-w

23 on behalf of the European Society of Radiology (ESR) and the European Society of Thoracic Imaging (ESTI), Revel M-P, Parkar AP, *et al.* COVID-19 patients and the radiology department – advice from the European Society of Radiology (ESR) and the European Society of Thoracic Imaging (ESTI). *Eur Radiol* 2020;**30**:4903–9. doi:10.1007/s00330-020-06865-y

24 Coleman JJ, Botkai A, Marson EJ, *et al.* Bringing into focus treatment limitation and DNACPR decisions: How COVID-19 has changed practice. *Resuscitation* 2020;**155**:172–9. doi:10.1016/j.resuscitation.2020.08.006

25 Briedé S, van Goor HMR, de Hond TAP, *et al.* Code status documentation at admission in COVID-19 patients: a descriptive cohort study. *BMJ Open* 2021;**11**:e050268. doi:10.1136/bmjopen-2021-050268

26 Brunn M, Kratz T, Padget M, *et al.* Why are there so many hospital beds in Germany? *Health Serv Manage Res* 2023;**36**:75–81. doi:10.1177/09514848221080691

27 Zangbar B, Rhee P, Pandit V, *et al.* Seasonal Variation in Emergency General Surgery. *Ann Surg* 2016;**263**:76–81. doi:10.1097/SLA.0000000000001238

28 Ljung R, Pihlström N, Sundström A. Risk of appendicitis after SARS-CoV-2 infection and SARS-CoV-2 vaccination. *Br J Surg* 2022;:znac127. doi:10.1093/bjs/znac127

For peer review only

FIGURE CAPTIONS

Figure 1: Number of patients admitted to the ICU with COVID-19 at the three hospitals, the changes in total cases of cholecystitis and the proportion treated with early surgery during the pandemic compared with 2017-2019.

For peer review only

For peer review only - http://bmjopen.bmj.com/site/about/guidelines.xhtml

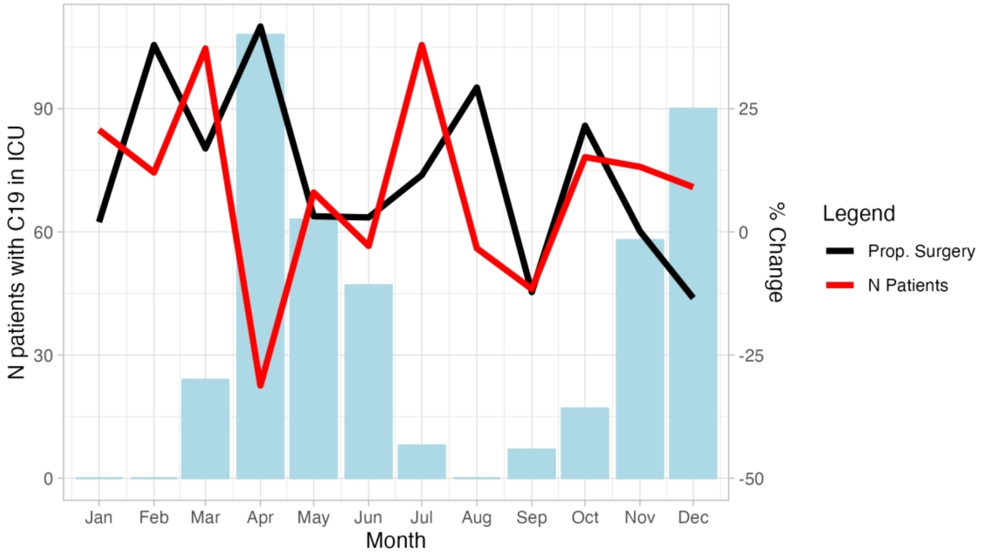

Figure 1: Number of patients admitted to the ICU with Covid-19 at the three hospitals and the changes in total cases of cholecystitis and the proportion treated with early surgery during the pandemic compared with 2017-2019.

177x101mm (300 x 300 DPI)

The RECORD statement – checklist of items, extended from the STROBE statement, that should be reported in observational studies using routinely collected health data.

	Item No.	STROBE items	Location in manuscript where items are reported	RECORD items	Location in manuscript where items are reported
**Title and abstract**					
	1	(a) Indicate the study's design with a commonly used term in the title or the abstract (b) Provide in the abstract an informative and balanced summary of what was done and what was found		RECORD 1.1: The type of data used should be specified in the title or abstract. When possible, the name of the databases used should be included.<br><br>RECORD 1.2: If applicable, the geographic region and timeframe within which the study took place should be reported in the title or abstract.<br><br>RECORD 1.3: If linkage between databases was conducted for the study, this should be clearly stated in the title or abstract.	1.1 Abstract: "Data was collected retrospectively from electronic patient records and the national registry for gallstone surgery"<br>1.2 "1,634 patients diagnosed with cholecystitis in two Swedish counties between 2017 and 2020 were included in the study."<br>1.3 See 1.1
**Introduction**					
Background rationale	2	Explain the scientific background and rationale for the investigation being reported			Background

For peer review only

Objectives	3	State specific objectives, including any prespecified hypotheses			Background second to last section
**Methods**					
Study Design	4	Present key elements of study design early in the paper			Method section 1
Setting	5	Describe the setting, locations, and relevant dates, including periods of recruitment, exposure, follow-up, and data collection			Method paragraph 2+3
Participants	6	*(a) Cohort study* - Give the eligibility criteria, and the sources and methods of selection of participants. Describe methods of follow-up<br>*Case-control study* - Give the eligibility criteria, and the sources and methods of case ascertainment and control selection. Give the rationale for the choice of cases and controls<br>*Cross-sectional study* - Give the eligibility criteria, and the sources and methods of selection of participants<br><br>*(b) Cohort study* - For matched studies, give matching criteria and number of exposed and unexposed<br>*Case-control study* - For matched studies, give matching criteria and the number of controls per case		RECORD 6.1: The methods of study population selection (such as codes or algorithms used to identify subjects) should be listed in detail. If this is not possible, an explanation should be provided.<br><br>RECORD 6.2: Any validation studies of the codes or algorithms used to select the population should be referenced. If validation was conducted for this study and not published elsewhere, detailed methods and results should be provided.<br><br>RECORD 6.3: If the study involved linkage of databases, consider use of a flow diagram or other graphical display to demonstrate the data linkage process, including the number of individuals with linked data at each stage.	6.1, 6.2, 6.3 Methods Reference to first paper describing the cohort.
Variables	7	Clearly define all outcomes, exposures, predictors, potential confounders, and effect		RECORD 7.1: A complete list of codes and algorithms used to classify exposures, outcomes, confounders, and effect modifiers should be provided. If	Method, Section 1 last paragraphs

For peer review only

		modifiers. Give diagnostic criteria, if applicable.		these cannot be reported, an explanation should be provided.	
Data sources/ measurement	8	For each variable of interest, give sources of data and details of methods of assessment (measurement).<br>Describe comparability of assessment methods if there is more than one group			Method, Reference to first paper with cohort
Bias	9	Describe any efforts to address potential sources of bias			Methods Reference to first paper with cohort
Study size	10	Explain how the study size was arrived at			Methods, All patients with diagnosis. Power analysis performed for first study. Power analysis reported in section Statistical analysis.
Quantitative variables	11	Explain how quantitative variables were handled in the analyses. If applicable, describe which groupings were chosen, and why			Methods, Section 1 paragraph 2 and reference to previous paper
Statistical methods	12	(a) Describe all statistical methods, including those used to control for confounding<br>(b) Describe any methods used to examine subgroups and interactions<br>(c) Explain how missing data were addressed<br>(d) *Cohort study* - If applicable, explain how loss to follow-up was addressed			a) Methods: Statistics, Paragraph 1<br>b) NA<br>c) Methods: Statistics, paragraph 1<br>d) Methods: Statistics,

For peer review only

		Case-control study - If applicable, explain how matching of cases and controls was addressed<br>*Cross-sectional study* - If applicable, describe analytical methods taking account of sampling strategy<br>(e) Describe any sensitivity analyses			paragraph 1<br>e) NA
Data access and cleaning methods		..		RECORD 12.1: Authors should describe the extent to which the investigators had access to the database population used to create the study population.<br><br>RECORD 12.2: Authors should provide information on the data cleaning methods used in the study.	12.2 Methods References first paper with cohort
Linkage		..		RECORD 12.3: State whether the study included person-level, institutional-level, or other data linkage across two or more databases. The methods of linkage and methods of linkage quality evaluation should be provided.	Methods, section 1, paragraph 2
**Results**					
Participants	13	(a) Report the numbers of individuals at each stage of the study (*e.g.*, numbers potentially eligible, examined for eligibility, confirmed eligible, included in the study, completing follow-up, and analysed)<br>(b) Give reasons for non-participation at each stage.<br>(c) Consider use of a flow diagram		RECORD 13.1: Describe in detail the selection of the persons included in the study (*i.e.,* study population selection) including filtering based on data quality, data availability and linkage. The selection of included persons can be described in the text and/or by means of the study flow diagram.	Methods section 1

For peer review only

Descriptive data	14	(a) Give characteristics of study participants (*e.g.*, demographic, clinical, social) and information on exposures and potential confounders<br>(b) Indicate the number of participants with missing data for each variable of interest<br>(c) *Cohort study* - summarise follow-up time (*e.g.*, average and total amount)			Results,<br>A+b) Table 1
Outcome data	15	*Cohort study* - Report numbers of outcome events or summary measures over time<br>*Case-control study* - Report numbers in each exposure category, or summary measures of exposure<br>*Cross-sectional study* - Report numbers of outcome events or summary measures			Results:<br>Temporal trends
Main results	16	(a) Give unadjusted estimates and, if applicable, confounder-adjusted estimates and their precision (e.g., 95% confidence interval). Make clear which confounders were adjusted for and why they were included<br>(b) Report category boundaries when continuous variables were categorized<br>(c) If relevant, consider translating estimates of relative risk into absolute risk for a meaningful time period			Results:<br>Delayed surgery, table 2<br>b) Done for period and otherwise NA<br>c) reported in "Delayed surgery and new gallstone complications" where applicable.
Other analyses	17	Report other analyses done—e.g., analyses of subgroups and			NA

For peer review only

		interactions, and sensitivity analyses			
**Discussion**					
Key results	18	Summarise key results with reference to study objectives			Discussion first few paragraphs
Limitations	19	Discuss limitations of the study, taking into account sources of potential bias or imprecision. Discuss both direction and magnitude of any potential bias		RECORD 19.1: Discuss the implications of using data that were not created or collected to answer the specific research question(s). Include discussion of misclassification bias, unmeasured confounding, missing data, and changing eligibility over time, as they pertain to the study being reported.	Discussion, Section Strengths and limitations
Interpretation	20	Give a cautious overall interpretation of results considering objectives, limitations, multiplicity of analyses, results from similar studies, and other relevant evidence			Discussion, first section paragraph 3-5
Generalisability	21	Discuss the generalisability (external validity) of the study results			Discussion, first section 5th paragraph
**Other Information**					
Funding	22	Give the source of funding and the role of the funders for the present study and, if applicable, for the original study on which the present article is based			Cover page
Accessibility of protocol, raw data, and programming code		..		RECORD 22.1: Authors should provide information on how to access any supplemental information such as the study protocol, raw data, or programming code.	Supplementary material and data availability

For peer review only

*Reference: Benchimol EI, Smeeth L, Guttmann A, Harron K, Moher D, Petersen I, Sørensen HT, von Elm E, Langan SM, the RECORD Working Committee. The REporting of studies Conducted using Observational Routinely-collected health Data (RECORD) Statement. *PLoS Medicine* 2015; in press.

*Checklist is protected under Creative Commons Attribution (CC BY) license.

For peer review only
